# Science Interest, Utility, Self-Efficacy, Identity, and Science Achievement Among High School Students: An Application of SEM Tree

**DOI:** 10.3389/fpsyg.2021.634120

**Published:** 2021-09-09

**Authors:** Amal Alhadabi

**Affiliations:** Kent State University, College of Education, Health, and Human Services, School of Foundations, Leadership, and Administration, Evaluation and Measurement, Kent, OH, United States

**Keywords:** science achievement, science identity, self-efficacy, science interest and science utility, SEM tree

## Abstract

The current study explored the associations between non–cognitive science-related variables, i.e., science interest, utility, self-efficacy, science identity, and science achievement in a serial mediation model. The study also further explored the potential heterogeneity in the model parameters using one of the data-mining techniques, which is the structural equation model (SEM) Tree. Data on 14,815 high school students were obtained from a large-scale database High School Longitudinal Study of 2009 (HSLS:09). The results highlighted science interest and science utility positively influencing science achievement through a sequential pathway of mediators, including science self-efficacy and science identity. The strength of direct effects considerably varied across students, resulting in classifying them into four subgroups. For instance, among females with a low SES subgroup, developing substantial science interest would result in better science self-efficacy and science identity that flourish science achievement. These valuable findings provide fruitful tailored recommendations, elevating the science achievement in the subgroups (146 words).

## Introduction

Science achievement among high school students correlates with the likelihood of enrollment in Science, Technology, Engineering, and Mathematics (STEM) majors during college. Many studies have acknowledged the role of cognitive abilities in shaping the science achievement of students (O'Reilly and McNamara, 2007). In contrast, other research has emphasized the role of non-cognitive qualities in scoring better academic grades, e.g., grit, self-efficacy, etc. (Alhadabi and Karpinski, 2019). A growing body of studies has found that science identity, as one of the non-cognitive attributes, had a proximal positive association with science achievement, e.g., Hill et al. (2018); Kim (2018); Williams et al. (2018). However, related works of literature have shown that students in the United States have been developing weak science identities and increased negative perceptions of science as a field of study, resulting in what is known as scientific pipeline leakage (Schultz et al., 2011). For example, the National Science Board (2016) showed that 17% more American students were at or below the 10% threshold of science achievement relative to other developed countries.

An extensive literature review has exhibited two main themes. The first theme refers to the vast individual differences found in science achievement, e.g., Aschbacher et al. (2010). This variability can be attributed to various factors. Demographics, including gender, socio-economic status (SES), and ethnicity, and non-cognitive science-related variables, including science utility, interest, self-efficacy, and identity, are potential explanatory factors for science achievement variability among high school students. Nonetheless, the literature has provided a mixed bag of findings of demographic information influencing science achievement. For example, while the majority of previous studies, e.g., Huang (2016); Kim and Sinatra (2018) showed that males were more likely to be efficient in science, the study of Vantieghem et al. (2014) clarified that feminists had a higher academic self-efficacy compared with boys. Contrarily, the literature has consistently demonstrated students with low SES and who belong to minority ethnic groups, such as Hispanic and African American, had a lower science achievement (Williams et al., 2018; Hanushek et al., 2019).

The second theme exhibited by related literature discusses an increasing number of qualitative studies stated that science identity and achievement are influenced by how students process information related to the self as a science person. For instance, the study of Archer et al. (2010) showed that holding high science self-efficacy, perceiving science utility, and investing effort reinforce a constructive science identity, which positively correlated with better science achievement (White et al., 2019). In contrast, a limited number of quantitative studies examined the association between non-cognitive science-related variables and science identity (Mohammadpour, 2013; Vincent-Ruz and Schunn, 2018; Alhadabi, 2020). None of these studies investigated the associations between the above-mentioned variables and science achievement.

As mentioned above, the findings outline various factors that lead to substantial variability in science achievement among high school students, including gender, SES, ethnicity, science interest, science utility, science self-efficacy, and science identity. However, the interaction between these factors has not yet been fully explored, and what categories of students are more likely to have the strongest associations between study variables and get higher science achievement. Structural Equation Model (SEM) tree is one of the novel and recently-developed statistical methods that can address the gap mentioned above and provide valuable answers (Brandmaier et al., 2013).

The reason for selecting this method can be attributed to the fact that SEM tree merges the parametric theory-driven, i.e., Structural Equation Modeling, and non-parametric data-driven, i.e., data mining techniques and particularly decision tree. Additionally, SEM Tree allows a simultaneous achievement of three tasks, which are: (1) modeling the associations between factors influencing science achievement using Path Analysis (PA) model, (2) selecting the most influential covariates in explaining the variability of model parameters, and (3) classifying the students by partitioning the data into homogenous groups/nodes in terms of model parameters, conditioning on the influential covariates (Brandmaier et al., 2014). Examples of questions that SEM tree can answer are: (1) Is the model investigating the associations between factors influencing science achievement fit the data?; (2) Is there significant variability in the model parameters, conditioning on the observed demographic covariates, i.e., gender, SES, and ethnicity?; (3) What are the most influential covariates?; (4) What are the splitting points that result in classifying students, and to what degree are these splits meaningful? Therefore, the objective of this study is to investigate the latent heterogeneity in the associations between the non-cognitive science-related variable and science achievement by answering the four questions mentioned above.

### Literature Review

Science achievement during high school, as one of the academic variables, identifies the enrollment of students in STEM majors. This seems a priority to meet the U.S. labor market needs (Radunzel et al., 2017). According to the U.S. Bureau of Labor Statistics (2020), employment in STEM occupations is projected to increase by 8% from 2019 to 2029. Even though opening jobs in the STEM field are growing, a well-known leaky STEM pipeline is expanding (Ahn et al., 2016). According to the study of Ahn et al. (2016), two reasons can explain this pipeline which leads to lower science achievement. First, the depersonalization of science content does not satisfy the need for the relatedness of students (Ryan and Deci, 2017). Second, students develop less attractive stereotypes and attitudes toward science and scientists that are exceptionally smart, invincible, confined to a laboratory setting, and detached from reality. This then leads to a repulsive reaction toward learning science, aligning with the study of Zhai et al. (2014).

A recent study found significant latent heterogeneity in student achievement among high school students, resulting in classifying students into three classes: (1) high-achieving class in which the academic achievement grew slightly across the 4 years of high school, (2) low-achieving class that showed a slight increase in the academic performance, and (3) moderate-achieving class that demonstrated a considerable decline in GPA across time (Alhadabi and Li, 2020). Further investigation of factors that can limit this pipeline and boost academic achievement among moderately and low achieving students is warranted. Many prior studies found that several non-cognitive variables resulted in higher science performance and alleviate such leaking in the scientific pipeline among high school, including science identity, science self-efficacy, science interest, and science utility (Archer et al., 2010; Alhadabi, 2020). This section provides a brief overview of these affective variables.

### Science Identity

Identity is expressed as the personal portrayal of students concerning their pursuits, beliefs, and anticipated accomplishments in a specific field (Hill et al., 2018). Therefore, science identity is the self-perception of being a science kind of person; in other words, someone who likes science is willing to invest the effort needed to be successful in pursuing a degree in science and is determined to accomplish their scientific goals (Archer et al., 2010). Psychological literature showed that identity construction is shaped by two processes which are crisis and commitment (Erikson, 1980). The crisis is a turning point where the adolescent is in active research and exploration of personal preferences. A state of continuous self-questioning results in knowing the self and developing the identity, e.g., Who am I? What is my current role? What shall I be in the future?. The second process, which is commitment expresses allegiance and adherence to the recognized preferences and selected roles.

The study of Marcia (1980) created a taxonomy, considering the extent of the crisis and commitment, i.e., low and high, resulting in new four states, i.e., diffusion, moratorium, foreclosure, and identity achievement. In the first state, diffusion, adolescents experience a low level of exploration and commitment. For example, adolescents are classified in a diffusion state when they do not recognize their skills and abilities, they do not apprehend the subjects that stimulate their curiosity, and they do not demonstrate dedication to something during middle and high school. On the other hand, students attain the identity moratorium by having active exploration and a weak sense of commitment. For instance, students explore various science subjects and participate and join different school clubs, yet they do not genuinely commit to any explored experiences and subjects. Identity foreclosure refers to showing high commitment to a specific field and is precisely imposed by significant others, e.g., parents, teachers, and peers; however, the student offers no or low active exploration of the imposed identity. Some students merely study STEM majors because their parents or older siblings are STEM persons, reflecting insufficient exploration and accepting imposed identity. The optimal state, identity achievement, is reached when students hold high exploration and commitment levels. The students clearly perceive themselves as science persons, show high dedication, and are viewed by others as science persons.

### Science Self-Efficacy

As one type of academic self-efficacy, science self-efficacy refers to the judgments of students about their abilities to successfully attain educational goals in science subjects (Elias and MacDonald, 2007). Students with high academic science self-efficacy are more likely to develop a more robust science identity, take more science courses, earn higher scores in these courses, and follow science career paths (Honicke and Broadbent, 2016; Stets et al., 2017). The related works of literature have demonstrated that high-efficacious students set high learning goals in a specific subject. As the value of goals increase, students invest more effort (Luszczynska and Schwarzer, 2005). A recent review of the literature on this topic found that academic self-efficacy is one of the mediators that play an intermediate part in the associations between the metacognitive, affective, and motivational regulation processes and academic achievement.

Related to the first two sets of regulation, the study conducted by Kirbulut and Uzuntiryaki-Kondakci (2018) revealed that science self-efficacy partially mediated the association between the meta-conceptual regulation, i.e., monitoring the existing conceptions and ideas, and the affective regulation, i.e., controlling and adopting the productive emotional state. Furthermore, it was a significant predictor of science achievement among eighth-grade students. Concerning the third set of actionable processes, i.e., motivation and achievement goals, a recent study found that academic self-efficacy mediated the relationship between the two dimensions of grit, i.e., perseverance of effort and consistency of interest, and three achievement orientation goals, i.e., mastery, approach, and avoidance goals, that had direct associations with academic performance (Alhadabi and Karpinski, 2019). This study emphasized that self-efficacy strengthens the positive effect of mastery and performance-approach goals and limits the negative impact of avoidance goals on academic performance. The study of Stets and colleagues (2017) exhibited a significant positive association between science identity and self-efficacy.

### Science Interest

Science interest reflects the cognitive potential of a student for achievement in the science field. The stronger the interest in science that a student has, the greater the commitment and effort to succeed. Studies have found that science interest among high school students is a strong predictor of enrolling in science-related courses and occupations (Hulleman and Harackiewicz, 2009). The study of Hazari et al. (2017) found that students who had higher science interest and studied in classrooms where their classmates shared the same high interest scored statistically higher STEM career intentions than other groups of students with a lower science interest. Furthermore, these students are more likely to adopt productive learning habits. That is, in the study of Singh et al. (2002), they found that students who have keen science interests spent more time doing homework and less time watching TV.

Nevertheless, a considerable body of literature has revealed a decline in science interest as students move from elementary to high school (George, 2006; Potvin and Hasni, 2014). In a research paper assessing the influence of grades (K-12) on the magnitude of science interest, the study of Greenfield (1997) showed that students in lower grades hold more interest in science relative to higher grades, indicating that high school students had a weaker interest in science. The study conducted by Osborne (2003) confirmed this decline during secondary schools, suggesting the investigation of the factor exacerbating such unconstructive attitudes toward studying science.

### Science Utility

Science utility pertains to the perception of a student regarding the importance of science as relevant or useful for the current and future goals at the individual and collective levels (Rozek et al., 2017). One of the psychological theories that explain how perceived science utility shapes the science outcomes is an expectancy-value theory (Eccles and Wigfield, 2002). That is, the value of any task, e.g., learning science, had four aspects which are (1) attainment value, i.e., the importance of learning science for the self-schema or identity of an individual; (2) intrinsic value, i.e., to what degree is learning science enjoyable; (3) utility value, i.e., the perceived usefulness and instrumental merits of science beyond the classroom; (4) cost, i.e., the perceived burden, sacrifices, and the price of learning science. Suppose the students hold an acute sense of the first three values, i.e., attainment, intrinsic, and utility, toward science. In that case, they are more likely to invest effort in learning science, diminishing the effect of the fourth type, i.e., cost value.

Findings showed contradictory conclusions related to the science utility growth across time. For instance, the findings in the study of George (2006) showed that the growth of science utility was positive as they moved through middle and high schools, contradicting the notable decline in the science interest (Potvin and Hasni, 2014). Other studies acknowledge the decline in the science utility, as projected directly by registering fewer or no science courses or indirectly by scarce involvement in science out-of-school activities, i.e., Simpkins et al. (2006). The study of Simpkins et al. (2006) found that students who hold stronger beliefs about their skills, capabilities, and interest in science are more likely to pursue studying science during adolescence than their peers. They found that participation in the science-related out-of-school activities at fifth-grade predicted task values and science utility at ninth-grade. A recently published quasi-experimental study revealed that students in the experimental group who received science utility value intervention had higher scores in science utility at the personal and communal levels compared with the control group (Shin et al., 2019).

### Study Model and Aim

Based on the extensive literature review mentioned above, a bigger picture reflects the need for examining the sequential order and influences of the non-cognitive science-related constructs, i.e., science interest, science utility, self-efficacy, and science identity on science achievement. Starting with distal variables and a more concrete perception of the inner propensity, keen curiosity and involvement, i.e., science interest, and valuing science instrumental merit, i.e., science utility, are not only cultivating science achievement indirectly (Singh et al., 2002; Simpkins et al., 2006; Hulleman and Harackiewicz, 2009). It also directly contributes to blossom the intermediate sense of the potential that is required to be successful as a science person, implying the role of science self-efficacy as a mediator, consistent with previous studies (Kirbulut and Uzuntiryaki-Kondakci, 2018; Alhadabi and Karpinski, 2019). As the level of the potential combined with the essential effort proliferate, a greater sense of science identity develops (Archer et al., 2010; Honicke and Broadbent, 2016; Stets et al., 2017). In turn, students who conceptualize themselves as science persons, i.e., science identity, and actualize this perception holding productive skills and potential, i.e., science self-efficacy would be more eager to perform well in science. Simultaneously, the associations between these variables are not static across the demographics of students, e.g., gender, SES, and ethnicity. For example, it is expected to have evident differences in the strength of association between these non-cognitive variables among males and females, aligning with prior studies (Banchefsky et al., 2016; Hill et al., 2018).

Furthermore, the current research acknowledges the importance of exploring the influences of school and the non-cognitive predictors of science teachers, e.g., science teaching self-efficacy, attitudes, and expectations about the performance of students, on the science achievement of students. However, the science teacher sample is not nationally representative in the current study data according to the High School Longitudinal Study of 2009 (HSLS:09) because of the low response rate (Ingels et al., 2011). Thus, the predictors of teachers were not examined in the current study.

**Figure 1:**
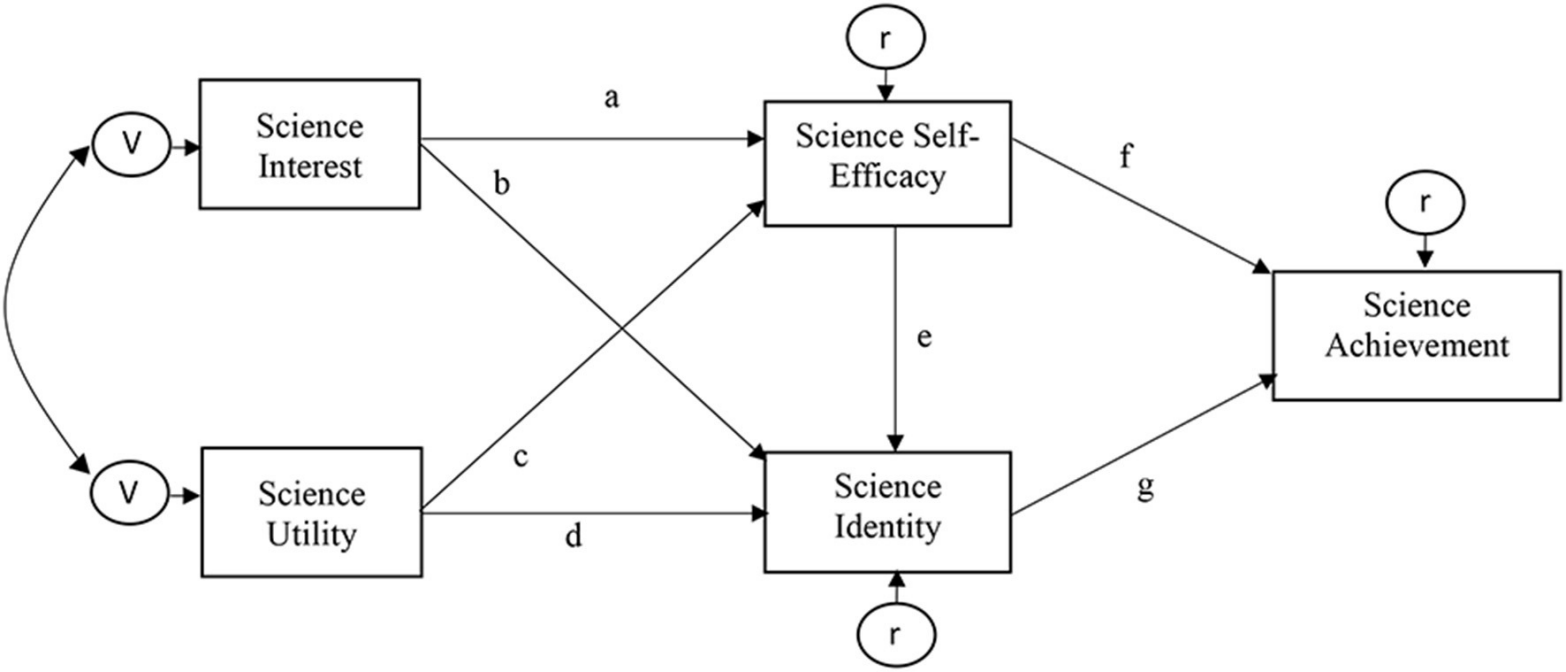
Conceptual model of the associations between science-related variables and science achievement. The double-headed curved arrow implies the covariance between the variance terms of the exogenous variables, which are, in this case, science utility and science interest. The right-to-left arrows and associated V in circles pointing toward science interest and utility are variance terms. The remaining three variables are endogenous, i.e., science self-efficacy, science identity, and science achievement. The right-to-left arrows and associated e in circles pointing toward these three variables are the residuals/unexplained variance terms of the endogenous variables due to error and other unmodeled variables.

This study achieved three research goals. It examined the associations between the non-cognitive science-related variables and science achievement among U.S. high school students using a serial mediation model. It also identified the most influential covariates, i.e., gender, SES, and ethnicity, in classifying students. After reviewing the literature, a conceptual model (see Figure 1) was examined using a sequence of direct and indirect hypotheses detailed below.

(H1) Keen science interest and science utility have positive direct associations with science self-efficacy, i.e., paths a and c, respectively, and science identity, i.e., paths b and d, respectively.

(H2) Science self-efficacy has a positive direct effect on science identity, i.e., path e, and science achievement, i.e., path f.

(H3) Constructive science identity is positively correlated with science achievement, i.e., path g.

(H4) Science interest and science utility have indirect effects on science achievement via science self-efficacy, i.e., paths a^*^f and c^*^f, and science identity, i.e., paths b^*^g and d^*^g.

## Method

### Participants

A complete sample of 14,815 U.S. high school students was obtained from the High School Longitudinal Study of 2009 (HSLS:09), particularly base-year data and 2013 update. This database was selected due to examining the factors influencing high school students' decisions regarding their future career paths, particularly STEM fields (Ingels et al., 2011). There were 7,284 (49.2%) males and 7,531 (50.8%) females. The majority of the students were white (*n* = 8,510; 57.4%). Other ethnic groups were Hispanic (*n* = 2,221; 15.0%), Asian (*n* = 1,254; 8.5%), African-American (*n* = 1,307; 8.8%), Hispanic with more than one race (*n* = 1,355; 9.1%), and very small number of other ethnic groups, e.g., Indian American and Native Hawaiian (*n* = 178; 1.2%).

### Measures

A survey was obtained from the student instrument administered in the base-year HSLS:09, containing six sections including (1) demographic information, i.e., gender, SES, and ethnicity; (2) science identity scale; (3) science self-efficacy scale; (4) science interest scale; (5) science utility scale; (6) science achievement as measured by grade point average for the highest science course that was taken in 2009 (GPA; Duprey et al., 2018).

The first measure, the science identity scale, was a two-item scale, i.e., “I see myself as a science person” and “Others see me as a science person”. Items are rated on a 4-point Likert scale ranging from “Strongly Agree” (Coded 1) to “Strongly Disagree” (Coded 4). The scale had good internal consistency reliability of α = 0.88 (Ingels et al., 2011).

The second measure, the science self-efficacy scale, has four items, reflecting the beliefs of students toward succeeding in science courses. Examples of the scale items are “I'm confident I can do an excellent job on fall 2009 science tests” and “I'm certain I can master skills in fall 2009 science course”. All items are rated on a 4-point Likert scale ranging from “Strongly Agree” (Coded 1) to “Strongly Disagree” (Coded 4). The scale demonstrated high-reliability consistency, i.e., Cronbach's α = 0.88.

The science interest scale consists of six items gauging the attitudes of students and their degree of interest in studying science, e.g., “I'm enjoying the fall 2009 science course very much” and “I think fall 2009 science course is a waste of time”. This scale also uses a 4-point Likert scale ranging from “Strongly Agree” (Coded 1) to “Strongly Disagree” (Coded 4). The reliability coefficient was high, i.e., Cronbach's α = 0.73.

The last scale, science utility, is a three-item scale rated on a 4-point Likert scale. Examples of the scale items are “I think the fall 2009 science course is useful for everyday life” and “I think fall 2009 science course is useful for future career”. The reliability coefficient was high (Cronbach's α = 0.75). The above-mentioned four non-cognitive variables, i.e., science identity, self-efficacy, utility, and interest, were derived from a principal component factor analysis. The composite values of scales were standardized with an *M* = 0 and an *SD* = 1 (Ingels et al., 2011).

### Data Analysis

Two primary analyses were conducted in the current study, namely Path Analysis (PA) and data mining using SEM tree. Several steps were followed to run the PA, i.e., Model specification, identification, estimation, testing, and modification. Related to the first step, the model was specified after reviewing the prior research, as explained in previous sections. The second step emphasizes the model should be over-identified or just-identified by comparing the number of parameters in the sample covariance matrix, i.e., S; it is a square matrix that estimates the covariance between each pair of data points, and model-implied covariance matrix, i.e., *Σ*; it is a square matrix that estimates the covariance values based on the proposed hypothetical model. Meaning, the number of distinct values in the matrix *S*, i.e., *k*(*k* + 1)/2, where *k* = the number of variables in the model, should be greater or equal to the number of free parameters estimated in the model (Schumacker and Lomax, 2016). This study has shown 15 distinct values in the matrix *S* and 11 free parameters, including the seven direct paths, three variance terms of the endogenous variables, i.e., science self-efficacy, science identity, science achievement, and one covariance term. The number of distinct values was greater than the free parameters, suggesting that the model is over-identified.

Related to the third step, a model estimation method is selected after examining the descriptive statistics for each variable. Demographic descriptive statistics were analyzed using R 4.0.2 (R Core Team, 2020). Data were screened before conducting the primary analyses, e.g., normality and outliers. Pearson correlations were computed. Based on the above information, Maximum Likelihood (ML) was selected as the estimation method (Schumacker and Lomax, 2016). Lavaan package was used to perform the PA (Rosseel, 2012) to specify the model, obtain the fit indices, and estimate the direct and indirect relationships.

Before model testing, the adequacy of the sample size was examined to ensure sufficient power and confidence in the Goodness-of-Fit (GoF) statistics. A minimum sample size of 100 to 200 is needed (Hoyle, 1995). A vast sample size was examined in the current study, which exceeds the recommendations made by the study of Hoyle. Several GoF indices were reviewed to assess the fit of the model to the data in the last step. Goodness-of-Fit were Chi-Square, Root-Mean-Square-Error of Approximation (RMSEA), Comparative Fit Index (CFI), Tucker-Lewis Index (TLI), and Standardized Root-Mean-Residual (SRMR).

In the second analysis, the SEM tree allows researchers to examine the fit of different template models, e.g., factor analysis, path analysis, latent class model, etc. Simultaneously, the dataset is recursively partitioned into nodes, i.e., groups, which maximally explain the differences in the outcome, i.e., model parameters, conditioning on influential observed covariates (Brandmaier et al., 2013). In other words, the SEM tree aims to identify the best split point or points that maximize the correct classification of the persons (Jacobucci et al., 2017), which results in maximally within-class homogeneity and maximum between-class heterogeneity. Using a dataset with two potential predictors, e.g., age and gender, SEM tree may partition the data based on one covariate or a combination of the two covariates, or none at all, corresponding to the strength of the associations between model parameters and the selected covariates (Brandmaier et al., 2016).

However, the SEM tree algorithm is a greedy recursive partitioning procedure (Brandmaier et al., 2013), resulting in a large number of nodes and an uninformative tree. Such nodes are highly unstable and cannot be generalized (Hayes et al., 2015). Different methods are suggested to control the depth of the SEM tree and assure the informativeness of nodes, which includes: (1) prespecifying constraints or customized stopping criteria; (2) applying the Bonferroni or cross-validation (cv) correction; (3) applying the maximum likelihood (ML) control methods or known as pruning techniques; (4) score-guided SEM tree.

The first method applies various constraints, e.g., a pre-specified number of nodes and a pre-specified number of participants per node (Brandmaier et al., 2014; Usami et al., 2017). These constraints are highly questionable in the literature (Hoyle, 1995). The second method controls multiple comparisons, i.e., these comparisons are used to identify the most significant covariate at each splitting point, inflated Type I error, and selection bias (Usami et al., 2017). The pruning techniques are alternative to stopping criteria, where the tree is allowed to grow as big as possible. A penalty is then applied to account for unstable and unnecessary nodes using cost-complexity pruning, resulting in a model with a sparer number of nodes (Hapfelmeier and Ulm, 2013). Four pruning methods include naïve, CV, fair, and fair3 (Hoyle, 1995; Brandmaier et al., 2014). Naïve does the split based on the value of the likelihood ratio test with a Bonferroni correction. As explained earlier, the CV method searches for the nodes in the training set and validates the results in the test set. Fair selects covariates with the highest response values. On the other hand, Fair3 is an extension of fair that retests all the split values.

The literature provides little guidance on which methods should be used. For instance, the study of Brandmaier et al. (2013) suggested the use of ML control methods, precisely fair and cv. However, the accuracy of subsequent splits is accounted for by the accuracy of the first split when using ML control methods (Grubinger et al., 2011). It means that failing to accurately estimate the first split results in a cumulative inaccuracy in the subsequent splits. Therefore, the score-guided SEM tree comes as a remedy for multiple ML comparisons by proposing an additional five methods. One simulation study found that two score-guided methods, i.e., maxLMO and CvM, outperformed ML methods in terms of statistical power, reducing computational time, and group recovery when examining multiple parameters (Arnold et al., 2021).

The current study identified the heterogeneity by examining multiple parameters, i.e., 11 direct effects, indirect effects, variance, and covariance terms. The majority of examined covariates which are gender, ethnic groups, and SES were dichotomous except SES. Therefore, an SEM tree package was used to construct the tree. The updated score-guided SEM tree, particularly maxLMO, controlled the tree depth (Arnold et al., 2021).

## Results

### Descriptive Statistics, Correlations, and Assumptions Checking

Table 1 tabulated the descriptive statistics. These statistics revealed no concern about normality violations. The presence of outliers was checked using the *z*-score method. That is, values of *z*-scores of all data points should be located between ± 2.58 along the normal curve. The *z*-scores which are > ± 2.58 imply probable outliers and values > ± 3.29 indicate extreme outliers (Field, 2009). In the current study, these values were lower than the conservative cutoff (*z* ± 2.58) and the liberal cutoff (*z* ± 3), implying no concern about the outliers. Table 2 showed that the hypothesized correlations were statistically significant.

**Table 1:** Descriptive statistics for the path analysis model variables (*N* = 14,815).

**Variables**	***M***	***SD***	**Min**	**Max**	**Skewness**	**Kurtosis**
Science achievement	2.58	0.97	0.25	4.00	−0.03	−2.00
Science identity	0.10	1.01	−1.57	2.15	0.08	−0.61
Science self-efficacy	0.06	0.99	−2.91	1.83	−0.24	0.38
Science interest	0.05	0.99	−2.59	2.03	−0.30	−0.18
Science utility	0.03	0.98	−3.10	1.69	−0.36	0.38

**Table 2:** Correlation coefficients for the path analysis model variables (*N* = 14,815).

**Variables**	**Achievement**	**Identity**	**Self-Efficacy**	**Interest**	**Utility**
1. Science achievement	–	0.19^***^	0.20^***^	0.08^**^	0.11^***^
2. Science identity		–	0.51^***^	0.41^***^	0.48^***^
3. Science self-efficacy			–	0.40^***^	0.52^***^
4. Science interest				–	0.51^***^
5. Science utility					–

** *p < 0.01*,

*** *p < 0.001*.

### Path Analysis Model

**Figure 2:**
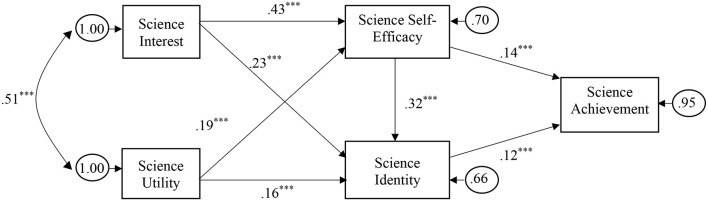
PA model with standardized parameters. The double-headed curved arrow implies the covariance between the variance terms of the exogenous variables, which are, in this case, science utility and science interest. The right-to-left arrows and associated figures in circles pointing toward science interest and utility are variance terms, which are estimated as 1, implying that the model did not explain the variances of these variables. The remaining three variables (i.e., science self-efficacy, science identity, and science achievement) are endogenous. The right-to-left arrows and associated figures in circles pointing toward these three variables are the residuals/unexplained variance terms of the endogenous variables due to error and other unmodeled variables. ^***^p < 0.001.

Results of the PA model demonstrated a significant Chi-square [χ^2^_(10)_ = 16,899.39, *p* < 0.001], indicating an unacceptable fit. This finding was reasonable because the sample size is enormous. The results of RMSEA, SRMR, CFI, and TLI were 0.00, 0.002, 1, and 1, respectively. These indices indicated a good model fit. All paths were significant and in the expected directions. The largest positive standardized path coefficients (see Figure 2) were between science interest and science self-efficacy (β = 0.43) and between science self-efficacy and science identity (β = 0.32). Compared with science interest, the utility had positive and weaker correlations with science self-efficacy and science identity. Science self-efficacy and science identity positively correlated with science achievement. Finally, there were eight significant indirect effects (see Table 3). The total effects of science interest and utility on GPA through science self-efficacy and science identity were significant. Overall, the serial mediation model was supported, including relationships hypothesized in H1 through H4.

**Table 3:** Final mediation model: maximum likelihood (standardized) estimates and selected fit indices.

**Description**	**Model parameters**	
	**Estimates**	***z*-values**
**Direct paths**		
Science interest → Science self-efficacy	0.43^***^	57.37
Science utility → Science self-efficacy	0.19^***^	23.64
Science self-efficacy → Science identity	0.32^***^	41.93
Science interest → Science identity	0.23^***^	27.81
Science utility → Science identity	0.16^***^	20.87
Science self-efficacy → Science achievement	0.14^***^	15.15
Science identity → Science achievement	0.12^***^	13.17
**Indirect paths**		
Science interest → Science self-efficacy → Science identity	0.14^***^	33.45
Science interest → Science self-efficacy → Science achievement	0.06^***^	14.58
Science interest → Science identity → Science achievement	0.03^***^	11.87
Science interest → Self-Efficacy → Identity → Science achievement	0.02^***^	12.20
Science utility → Science self-efficacy → Science identity	0.06^***^	20.56
Science utility → Science self-efficacy → Science achievement	0.03^***^	12.72
Science utility → Science identity → Science achievement	0.02^***^	11.11
Science utility → Self-Efficacy → Identity → Science achievement	0.01^***^	11.05
**Total effect**		
Science interest → Science achievement	0.24^***^	38.33
Science utility → Science achievement	0.11^***^	24.05
**Selected fit indices**		
*X^2^*	16,599.39^***^	
Root-Mean square error of approximation (RMSEA)	0.02, CI (0.01–0.03)	
Standardized root-mean-square residual (SRMR)	0.01	
Comparative fit index (CFI)	1.00	
Tucker-Lewis index (TLI)	1.00	

*** *p < 0.001*.

### SEM Tree Findings

**Figure 3:**
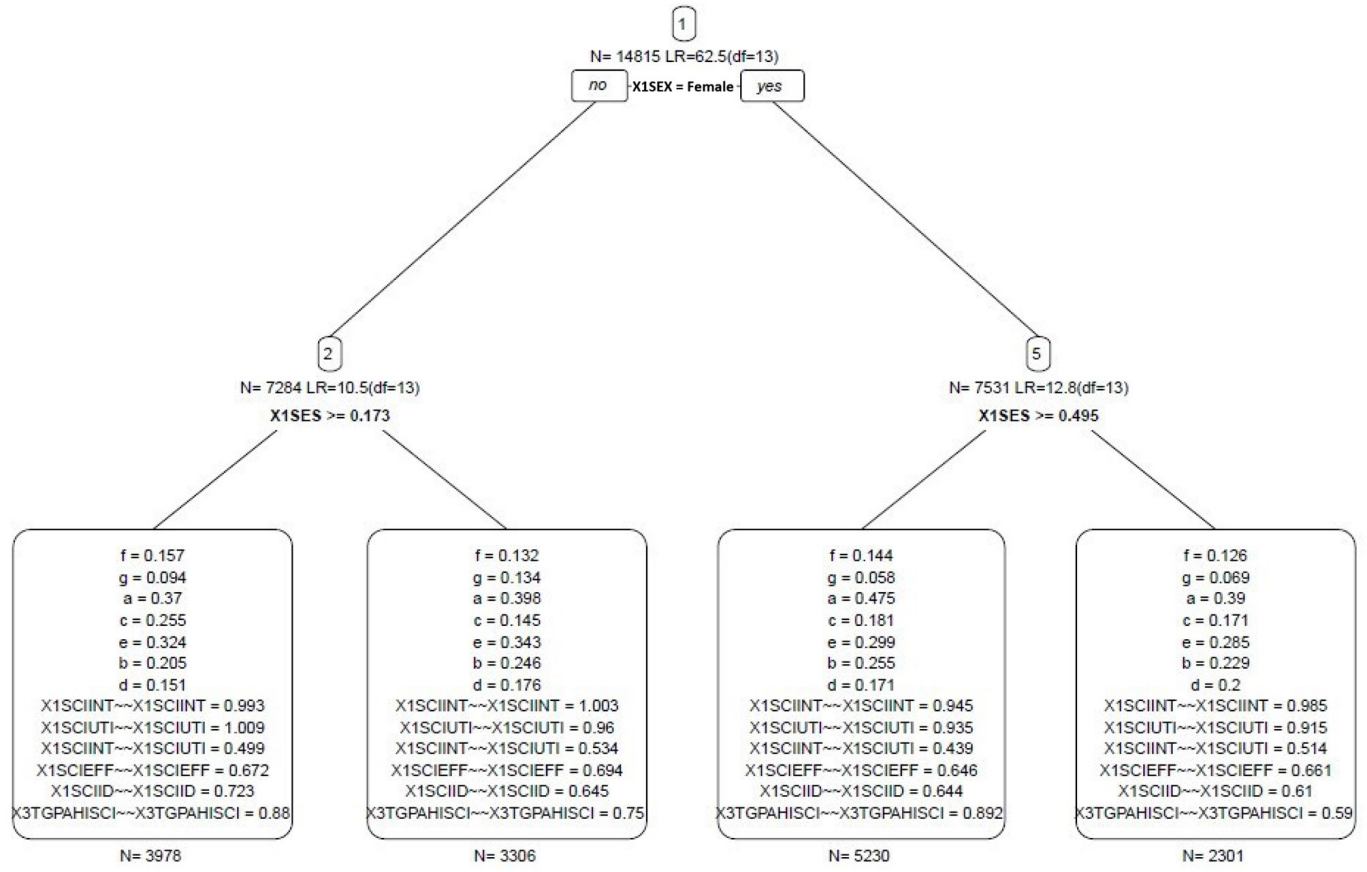
SEM tree model. a = Science Interest → Self-Efficacy, b = Science Interest → Science Identity, c = Science Utility → Science Self-Efficacy, d = Science Utility → Science Identity, e = Science Self-Efficacy → Science Identity, f = Self-Efficacy → Science Achievement, g = Science Identity → Science Achievement, ~~ = variance.

The findings in the SEM tree identified that gender and SES were the most influential covariates in classifying students based on the heterogeneity of model parameters (see Figure 3). The tree resulted in three splitting points and four nodes. The most influential covariate was gender, resulting in classifying students into male and female nodes. SES was the most influential covariate among males, creating two nodes (i.e., low and high SES nodes of males). That is students with low SES, i.e., SES <0.17, had relatively lower direct paths between studied variables compared with males with higher SES (see Table 4), except for paths f, i.e., self-efficacy to achievement, and c, i.e., utility to self-efficacy. Meaning, males with low SES who had higher science utility were more likely to report stronger self-efficacy, and these students with stronger self-efficacy were associated with a better GPA.

**Table 4:** PA directs effects estimates for the subgroups derived from the SEM tree.

**Description**	**Males (** ***n*** **=** **7,284)**		**Female (** ***n*** **=** **7,531)**	
	**Low SES** **(*n* = 3,978)**	**High SES** **(*n* = 3,306)**	**Low SES** **(*n* = 5,230)**	**High SES** **(*n* = 2,301)**
**Direct paths**				
Path a = Science interest → Science self-efficacy	0.37^***^	0.40^***^	0.48^***^	0.39^***^
Path c = Science utility → Science self-efficacy	0.26^***^	0.15^***^	0.18^***^	0.17^***^
Path e = Science self-efficacy → Science identity	0.32^***^	0.34^***^	0.30^***^	0.29^***^
Path b = Science interest → Science identity	0.21^***^	0.25^***^	0.26^***^	0.23^***^
Path d = Science utility → Science identity	0.15^***^	0.18^***^	0.17^***^	0.20^***^
Path f = Self-Efficacy → Science achievement	0.16^***^	0.13^***^	0.14^***^	0.13^***^
Path g = Science identity → Science achievement	0.09^***^	0.13^***^	0.06^***^	0.07^***^

*** *p < 0.001*.

Comparatively, SES was the most influential covariate among females. Unlike male nodes, female students with low SES, i.e., SES < 0.50, had relatively stronger direct effects between studied variables than females with higher SES. For example, the direct association between science interest and science self-efficacy among females with low SES (β = 0.48) was higher than that among females with high SES (β = 0.39). It implies that developing science-related non-cognitive variables would have a greater impact on females with low SES. None of the ethnic groups were significantly influential in classifying students.

## Discussion

Even though the projected demand for STEM jobs is growing (U.S. Bureau of Labor Statistics, 2020), a well-documented scientific pipeline reflects an increasing propensity to avoid learning science, resulting in low science achievement and low STEM enrollment (Schultz et al., 2011). Yet, considerable heterogeneity has been observed in the academic performance of high school students (Alhadabi and Li, 2020), encouraging exploring the most influential covariates in such variability, precisely science achievement. An increasing body of studies acknowledged the diversity of factors influencing academic achievement and student enrollment in STEM fields, e.g., demographic information, cognitive skills, and affective qualities (O'Reilly and McNamara, 2007; Aschbacher et al., 2010). Greater emphasis has been placed on science-related non-cognitive characteristics, e.g., scientific interest, science utility, science self-efficacy, and science identity. None of these variables has been examined simultaneously along with science achievement. The heterogeneity in associations between these qualities and science achievement has not been explored yet, conditioning the demographic information using data mining techniques. Taking this further, the current study hypothesized a path model derived from a literature review (see Figure 1). Additionally, it explored the heterogeneity of model parameters using one of the very recent methodological techniques, SEM tree.

The model illustrated a good fit and showed significant direct and indirect effects. In the left part of the model, science interest had the strongest positive correlation with science self-efficacy and a relatively weaker positive association with science identity. Students with higher science interests had higher science self-efficacy and adopted a stronger belief in science identity. These findings imply students who hold genuine interest would invest more effort, regulate their studying science habits like doing more science homework, out-of-school activities, taking more science courses, and watching less TV (Singh et al., 2002; Simpkins et al., 2006; Hulleman and Harackiewicz, 2009). Science utility also positively correlated with science self-efficacy and science identity, substantiating the expectancy-value theory (Eccles and Wigfield, 2002). In other words, the perceptions of students on science utility empower their sense of self-schema as science persons and strengthen their abilities to attain education goals in science (Stets et al., 2017). The current study revealed the strength of these influences differed across the science interest and utility. Notably, science interests had stronger associations with science self-efficacy and identity compared with science utility, suggesting more attention and focused intervention should establish to reinforce the science interest of students.

In the intermediate part of the model, the findings showed that science self-efficacy had direct positive effects on science identity and science achievement, supporting the constructive role of self-efficacy. Again, science self-efficacy mediated the association between two variables on the left side of the model, i.e., science interest and utility, and science identity. These findings extend the findings of previous studies (Kirbulut and Uzuntiryaki-Kondakci, 2018; Alhadabi and Karpinski, 2019). In other words, science self-efficacy strengthens the association between affective attachment and keen involvement in science, i.e., science interest, and science identity. Concurrently, self-efficacy nourishes the relationship between the conceptual perception of science usefulness, i.e., science utility, and identity. Furthermore, science identity itself had a significant direct positive effect on science achievement, in good agreement with the study conducted by Williams et al. (2018).

Related to the serial mediation hypotheses, the findings showed that science interest and utility had positive indirect effects on science identity via self-efficacy. Furthermore, they had indirect effects on science achievement via science identity and science self-efficacy. The findings lend support reasonably well to the qualitative research line, which outlined that science identity and achievement are influenced by how students process information related to themselves as a science person, their abilities, and science usefulness (Archer et al., 2010; White et al., 2019). These findings provide a theoretical framework for the direct and indirect associations between non-cognitive variables and science achievement, supporting affective valuable contributions of science-related characteristics. The framework adds new evidence and expands the limited quantitative studies (Mohammadpour, 2013; Vincent-Ruz and Schunn, 2018; Alhadabi, 2020).

Related to the novel and new data mining algorithm, this study revealed considerable heterogeneity in the model parameters among students. Gender was the most influential covariate that did the first split in the data, resulting in two nodes, i.e., women and men. Socio-economic status was the second significant covariates with different critical values in the nodes of males and females. That is, the SES critical value was (*t* = 0.17) in male node, and (*t* = 0.50) in the female node. None of the ethnic groups were significantly influential in classifying students.

As a result, students were classified into four nodes/subgroups based on the variability of the model parameters conditioning on two observed covariates. These nodes are (1) males with low SES, (2) males with high SES, (3) females with low SES, and (4) females with high SES. The strength of the associations between the studied variables varied across the four nodes. For example, the association between science interest and science self-efficacy had the strongest coefficient (β = 0.48) among female students with low SES. This finding implies that developing science interest would flourish the science self-efficacy of female students with low SES much better compared with other students, aligning with the study of Vantieghem et al. (2014). On the other hand, the associations among males between science interest and science self-efficacy had the strongest coefficient (β = 0.40) among male students with high SES.

In a nutshell, across the female nodes, female students with low SES had relatively stronger associations between studied variables than females with higher SES. It suggests that developing science-related non-cognitive variables would significantly impact science achievement among females with low SES. Across male nodes, students with low SES had relatively lower direct paths between studied variables than males with higher SES, except for paths f, i.e., self-efficacy to achievement, and c, i.e., utility to self-efficacy. Meaning, conducting interventions to empower science self-efficacy and utility would enhance science achievements among males with low SES.

### Implications and Limitations

The findings of this study have fruitful practical applications. Educators and school administrators may help students perform academically better in science by strengthening their science interest, utility, self-efficacy, and identity. That is, educators can design interventions that promote a learning atmosphere, which develops affective science-related characteristics. These interventions can be tailored according to the needs of student groups. For instance, men with low SES students would mainly benefit from interventions that focus on developing science self-efficacy and science utility. For women with low SES, enhancing science interest and science self-efficacy can be more productive. Yet, the current study also has limitations. The study examined demographic student-related covariates. Family-related and school-related covariates would provide more in-depth insights into the latent variability in the model parameters influencing science achievement.

### Conclusion

In conclusion, the prosperity and advancement of the future are deeply rooted in the STEM field. The goal of all nations, including the U.S., is to boost science achievement among high school students, further improving enrollment in STEM majors at the collegiate level. Elevating the STEM enrollment is not the ultimate end, but improving the quality and impact of future scientific discoveries and advancement are far more critical. However, substantial variability is apparent in the academic science achievement among high school students. This variability can be attributed to several factors. Among these factors, non-cognitive science-related constructs have significant influences on science achievement, i.e., science interest, science utility, self-efficacy, and science identity. That is, students who conceptualize themselves as science persons, i.e., science identity, and actualize this perception by holding productive skills and potential, i.e., science self-efficacy, would be more eager to perform well in science. Simultaneously, the inner beliefs are not isolated from a broader and more tangible perception of science instrumental merit, i.e., science utility, and keen curiosity and involvement, i.e., science interest. The associations between these variables are not static across the demographics of students. Identifying the most influential covariates that are conditioned on these associations offers tailored recommendations that would constructively flourish science achievement for each group.

## Data Availability Statement

The original contributions presented in the study are included in the article/supplementary material, further inquiries can be directed to the corresponding author.

## Ethics Statement

Ethical review and approval was not required for the study on human participants in accordance with the local legislation and institutional requirements. Written informed consent to participate in this study was provided by the participants' legal guardian/next of kin.

## Author Contributions

AA prepared the research starting from identifying the problem, stating the research questions, reviewing the literature, obtaining the data, conducting the analyses, and interpreting the results.

## Conflict of Interest

The author declares that the research was conducted in the absence of any commercial or financial relationships that could be construed as a potential conflict of interest.

## Publisher's Note

All claims expressed in this article are solely those of the authors and do not necessarily represent those of their affiliated organizations, or those of the publisher, the editors and the reviewers. Any product that may be evaluated in this article, or claim that may be made by its manufacturer, is not guaranteed or endorsed by the publisher.
